# Profile and professional expectations of medical students in Mozambique: a longitudinal study

**DOI:** 10.1186/1478-4491-8-21

**Published:** 2010-09-21

**Authors:** Paulo Ferrinho, Inês Fronteira, Mohsin Sidat, Fernando da Sousa, Gilles Dussault

**Affiliations:** 1 Associação para o Desenvolvimento e Cooperação Garcia de Orta (AGO), Lisbon, Portugal; 2 Unidade de Sistemas de Saúde e Centro de Malária e Outras Doenças Tropicais, Instituto de Higiene e Medicina Tropical, Universidade Nova de Lisboa, Lisbon, Portugal; 3 Faculty of Medicine, University Eduardo Mondlane, Maputo, Mozambique

## Abstract

**Introduction:**

This paper compares the socioeconomic profile of medical students registered at the Faculty of Medicine of Universidade Eduardo Mondlane (FM-UEM), Maputo, for the years 1998/99 and 2007/08.

**Case study:**

The objective is to describe the medical students' social and geographical origins, expectations and perceived difficulties regarding their education and professional future. Data were collected through questionnaires administered to all medical students.

**Discussion and evaluation:**

The response rate in 1998/99 was 51% (227/441) and 50% in 2007/08 (484/968).

The main results reflect a doubling of the number of students enrolled for medical studies at the FM-UEM, associated with improved student performance (as reflected by failure rates). Nevertheless, satisfaction with the training received remains low and, now as before, students still identify lack of access to books or learning technology and inadequate teacher preparedness as major problems.

**Conclusions:**

There is a high level of commitment to public sector service. However, students, as future doctors, have very high salary expectations that will not be met by current public sector salary scales. This is reflected in an increasing degree of orientation to double sector employment after graduation.

## Introduction

In Mozambique, medical students are trained in three faculties: two are public institutions (Faculty of Medicine of the University Eduardo Mondlane (MF-UEM), in Maputo, and Faculty of Health Sciences of Lúrio University, in Nampula) and the Catholic University in Beira, a private institution. The Faculty of Medicine in Beira, functioning since 2000, produced its first graduates in 2007. The Nampula faculty started in 2007. The joint output of graduates is currently approximately 100 medical doctors per year, with a total of 817 doctors having graduated from FM-UEM between 1975 and 2007. In addition, about 100 doctors were trained in foreign countries, mostly in Cuba [1].

A previous study of medical students registered for the 1998/99 academic year in the MF-UEM, showed that academic performance was poor, which students explained by lack of library facilities, inadequate financial support, and poor high school preparation. Students knew that they would be needed in the public sector, and that this represented an opportunity to contribute to the welfare of the population. Nevertheless, their expectations were to combine public sector practice with private medical work in order to improve their earnings [2].

This new paper compares the profile and expectations of medical students from the 2007/08 academic year at UEM with those of 1998/99.

## Case description

The methodology for the 1998/99 study, as previously described [2], is similar to the one adopted for this most recent study. In 2007/2008, a piloted, standardized questionnaire, with closed and open-ended questions, was distributed to all registered medical students (1^st ^to 7^th ^year of medical education). The anonymous questionnaires, pre-tested among staff of the community health department of the faculty, were distributed and collected by a member of the students' association. The questions addressed sociodemographic characteristics; reasons for choosing medicine as a profession; difficulties regarding the learning process in the medical school; and professional and salary expectations after leaving medical school. Some of the questions were similar to the ones applied in the previous study and these are the ones analysed in this paper.

The study 'universe' (i.e. the number of students registered for medical education) at UEM in 2007/08 was more than double the universe of 1998/99. The response rate in 1998/99 was 51% (227/441) and 50% in 2007/08 (484/968) and these two populations are compared in Table 1. Although similar in age, there is a lower percentage of females in the most recent study.

**Table 1:** Profile of study populations, 1998/99 (N = 227), 2008/09 (N = 484)

		1998/1999	2007/2008
Age in years	Mean	23	23
	Range	18-36	17-43
Gender	Female	61%	50%
Marital status	Not married	90%	91%
Place of birth	Maputo city or Province	44%	52%
Completed primary school	Maputo city or Province	44%	58%
Secondary school	Maputo city or Province	63%	59%
Age of decision to study medicine	< 18	65%	83%

Data were entered in a Microsoft Access database and analysed with SPSS 17.0.

## Discussion and evaluation

### Students' background

In both studies, a significant proportion of students was born and received primary school education outside Maputo Province and Maputo City, where the medical school is located. On the other hand, most of the students enrolled in the medical school completed high school education in Maputo (city or province), although these regions are only home to about 11.4% (6% for Maputo Province and 5.4% for Maputo City) of the country's population. But these patterns are less marked in the recent study, probably reflecting the ability of the other faculties of medicine, outside Maputo, to absorb candidates from other provinces, leaving UEM a greater degree of freedom to concentrate its intake in and around Maputo (Table 1).

### The decision to study medicine

Both groups of students took the decision to study medicine when they were in their late teens (Table 1).

The main reasons to choose medicine as a profession were "to contribute to the welfare of the public" (60% in 1998/99 vs. 37% in 2007/08), "self-achievement" (48% in 1998/99 vs. 28% in 2007/08), "vocation" (34% in 1998/99 vs. 23% in 2007/08) and "social recognition" (13% in 1998/99 vs. 2% in 2007/08). A reason mentioned by 2% of the students in 2007/08 but not in 1998/99 was that medicine as a profession opened possibilities of a stable job market. The disparity between the two set of replies might be understood if we consider that in 1998 the question options were closed, but open-ended in 2007/2008. Even so, the ranking order of the reasons presented is similar for the two studies.

In 1998/99, 90% of the students reported that their parents had in some way been associated with the health sector: as doctors (29%), nurses (29%), other health sector personnel (18%), pharmacists (8%), and auxiliaries (2%) or in some other category (5%). Furthermore, 46% reported having uncles and/or aunts that were associated with the health profession, with 24% having friends working in the discipline and 30% noting other reference people similarly involved. In 2007/08, data were collected in a different format (open ended question in 1998/99 and question with closed options in 2007/08) and 45% of the students indicated that their relatives did not have any influence on their decision to choose medicine (only 38% of them had one or more relatives in the health professions); 30% acknowledged a strong influence (53% had relatives in the health professions); and 25% some but weak influence (48% had relatives in the health professions).

### Academic performance

In 1998/99, 6% of the 79 first year students were repeating the year for the second or third time. Only 32% of the 143 students enrolled in the subsequent years had not failed any academic year. In 2007/2008, 8% of 121 first year students were repeating the year. Although 92% of the students had not failed any academic year, 20% reported passing to the next academic year while not having completed the syllabus of previous years.

### Main difficulties reported

The most frequent difficulties reported (these are not mutually exclusive) by students surveyed in 1998/99 were: "lack of reference materials/literature" (66% of students), and "financial problems" (58%). Other difficulties were "lack of adequate technology" (22%), "teachers not adequately prepared" (22%), "inadequate syllabus" (8%) and "inadequate preparedness by the high school education system" (8%).

In 2007/2008 the main reasons for dissatisfaction with the available support systems within the faculty were associated with the library (55%), the computer room (44%), with the lack of learning equipment (18%) and with the lack of laboratory support (14%).

### Satisfaction with education received

In 1998/99, 54% of the students were satisfied or partially satisfied with the burden of lecturing and learning hours demanded by the medical school; 26% were unhappy or partially unhappy with it and 20% did not have any opinion. Regarding the quality of the training received 52% felt it was adequate or very adequate, and 20% that it was inadequate or very inadequate and the balance did not have any opinion.

In 2007/2008, 29% of the students were satisfied or partially satisfied with the burden of lecturing and learning hours demanded by the medical school; 36% were unhappy or partially unhappy with it and 35% were neither satisfied nor unsatisfied.

### Expectations regarding professional activities and income

When asked about their intentions regarding the sectors where they would like to practice medicine after completing their medical education (more than one choice possible), the proportion of students interested in double sector employment increased from 53% in 1998/99 to 78% in 2007/2008.

Concerning what they expect as monthly income upon graduation, the choices are presented in Table 2, reflecting a steep increase in salary expectations over the last 10 years, much above public sector salary scales.

**Table 2:** Income expectations of medical students after graduation (exchange rate +26 meticais for USD)

Income (meticais)		1998/99		2007/08	
		N	%	N	%
Less than 15 000		105	49.3	122	26.5
15 000 to 19 999		37	17.4	82	17.9
20 000 or more		71	33.3	251	54.6
Total		213	100.0	460	100.0
	1st year of practice as an intern	3345 meticais		8617 meticais	
Reference salaries					
	Top salary for public sector doctor	6488 meticais		16 712 meticais	

## Conclusions

Because of methodological difficulties associated with the differing formulation of the questions in the two studies under comparison, we do not expect our study to be generalizable. Although not generalizable, it still provides information and lessons that might be useful, not only to the Maputo Faculty, but also to other Mozambican and African Faculties. The studies provide longitudinal data about medical training in Africa, more specifically in Mozambique, a theme that has received little coverage in the literature.

In spite of the dismal student performance observed in the past (reflected as failure rates), a problem also described regarding other African medical faculties [1,3-9] this recent study is associated with improved student performance, a not insignificant achievement in the context where the number of students enrolled for medical studies at the FM-UEM doubled.

Nevertheless, satisfaction with the training received remains low. Now as before--and similarly to medical students in Angola and Guinea-Bissau--students still identify lack of access to books or learning technology and inadequate teacher preparedness as major problems [1]. Nevertheless, students remain confident that the training received will allow them to be good, competent doctors in Mozambique and elsewhere [10].

As for medical students in other Portuguese speaking African countries [11], there is a high level of commitment to public sector service. However, students, as future doctors, have very high salary expectations that will not be met by current public sector salary scales, a finding similar to the expectations of medical students in Guinea-Bissau [1]. This is reflected in an increasing degree of orientation to double sector employment after graduation.

## Competing interests

The authors declare that they have no competing interests.

## Authors' contributions

PF wrote the protocol and questionnaire, coordinated both studies and wrote the initial manuscript. IF carried out the statistical analysis. MS conducted the field work in 2007/8. FS conducted the field work in 1998/9. GD helped to conceptualize the paper and commented on all drafts. All authors read and approved the final manuscript
